# Exploring the Genetic Landscape of Retinal Diseases in North-Western Pakistan Reveals a High Degree of Autozygosity and a Prevalent Founder Mutation in *ABCA4*

**DOI:** 10.3390/genes11010012

**Published:** 2019-12-21

**Authors:** Atta Ur Rehman, Virginie G. Peter, Mathieu Quinodoz, Abdur Rashid, Syed Akhtar Khan, Andrea Superti-Furga, Carlo Rivolta

**Affiliations:** 1 Division of Genetic Medicine, Lausanne University Hospital and University of Lausanne, 1011 Lausanne, Switzerland; 2 Institute of Experimental Pathology, Lausanne University Hospital and University of Lausanne, 1011 Lausanne, Switzerland; 3 Department of Genetics and Genome Biology, University of Leicester, Leicester LE1 7RH, UK; 4 Clinical Research Center, Institute of Molecular and Clinical Ophthalmology Basel (IOB), 4031 Basel, Switzerland; 5 Department of Computational Biology, University of Lausanne, 1015 Lausanne, Switzerland; 6 Government Degree College Ara Khel, FR Kohat 26000, Khyber Pakhtunkhwa, Pakistan; 7 Department of Ophthalmology, Khalifa Gul Nawaz Hospital, Bannu 28100, Pakistan; 8 Department of Ophthalmology, University Hospital Basel, 4031 Basel, Switzerland

**Keywords:** hereditary retinal diseases, autozygosity mapping, consanguinity, Pakistan

## Abstract

Variants in more than 271 different genes have been linked to hereditary retinal diseases, making comprehensive genomic approaches mandatory for accurate diagnosis. We explored the genetic landscape of retinal disorders in consanguineous families from North-Western Pakistan, harboring a population of approximately 35 million inhabitants that remains relatively isolated and highly inbred (~50% consanguinity). We leveraged on the high degree of consanguinity by applying genome-wide high-density single-nucleotide polymorphism (SNP) genotyping followed by targeted Sanger sequencing of candidate gene(s) lying inside autozygous intervals. In addition, we performed whole-exome sequencing (WES) on at least one proband per family. We identified 7 known and 4 novel variants in a total of 10 genes (*ABCA4*, *BBS2*, *CNGA1*, *CNGA3*, *CNGB3*, *MKKS*, *NMNAT1*, *PDE6B*, *RPE65*, and *TULP1*) previously known to cause inherited retinal diseases. In spite of all families being consanguineous, compound heterozygosity was detected in one family. All homozygous pathogenic variants resided in autozygous intervals ≥2.0 Mb in size. Putative founder variants were observed in the *ABCA4* (NM_000350.2:c.214G>A; p.Gly72Arg; ten families) and *NMNAT1* genes (NM_022787.3:c.25G>A; p.Val9Met; two families). We conclude that geographic isolation and sociocultural tradition of intrafamilial mating in North-Western Pakistan favor both the clinical manifestation of rare “generic” variants and the prevalence of founder mutations.

## 1. Introduction

Inherited retinal dystrophies (IRDs) constitute a genetically heterogeneous group of rare conditions of the eye. They are mainly characterized by the progressive loss of rod and/or cone photoreceptors, resulting in complete or nearly complete blindness at the end [1]. Globally, IRDs affect approximately one million people, with a frequency of 1 in 3000 births. Clinically, they may range from mild and non-progressive night blindness to more severe and degenerative phenotypes, including retinitis pigmentosa (RP) and cone or cone-rod dystrophies [2]. To date, mutations in over 271 genes have been linked to various forms of IRDs (RetNet; https://sph.uth.edu/RETNET/; accessed on 12 December 2019), and the sequencing of their coding parts has allowed the detection of pathogenic mutations in more than 60% of the patients [3]. IRDs are inherited as an autosomal recessive, autosomal dominant, X-linked, or mitochondrial trait, with autosomal recessive being the most prominent type [1,4]. Recent technological advancements, such as next-generation sequencing (NGS), have significantly increased gene discovery rates in a wide range of inherited ocular conditions [5], with a sensitivity value of ~75%, when applied to a clinically focused IRDs group [6]. Since consanguinity unmasks the adverse effects of recessive mutations through bi-parental inheritance of the same allele, it is possible to reveal the presence of disease-causing variants in consanguineous pedigrees by simply flagging large segments of consecutive homozygous genotypes surrounding the mutations, using a technique called “autozygosity mapping” [7,8,9,10]. For a more rapid and robust analysis, scientists usually combine autozygosity mapping with NGS to maximize the acquisition of relevant genetic information [10]. The combination of such information with targeted functional studies has provided significant insights into the molecular mechanisms of rare Mendelian diseases, including IRDs.

Since children of consanguineous couples are more likely than children of non-consanguineous parents to be affected by recessive genetic anomalies [11], the incidence of rare Mendelian diseases is higher in populations having a high degree of endogamy [12]. For example, Pakistan has one of the highest rates of inherited genetic diseases in the world, likely due to the fact that consanguinity is present in more than 50% of the population and marriages among first cousins are highly favored by the society [13,14]. According to a recent estimate, approximately 1.12 million people in Pakistan are blind, and the vision loss burden has continued to rise in the country since 1990 [15].

Although some information exists on blindness caused by cataracts or refractive errors, the prevalence of IRDs is not well documented in Pakistan at the level of the whole population. A hospital-based study in Karachi, a metropolitan city, revealed that 1 in 800 patients who visited the ophthalmic outpatient department had retinal dystrophies, with RP being the most frequent type (64%), followed by Stargardt disease (14.7%) and cone dystrophies (6.7%). Unsurprisingly, more than half of the patients from this study were born to consanguineous parents [16]. Recently, a few studies on IRDs have been published in Pakistan [17,18,19,20]. However, the majority of these reports were based on pedigrees from the Punjab and Sindh provinces, thus leaving North-Western Pakistan largely unexplored. Administratively known as Khyber Pakhtunkhwa (KP), this part of the country is predominantly a Pashtuns territory and includes a heterogeneous population of approximately 35 million inhabitants. Consanguinity in KP ranges between 22% and 66%, and the rate of consanguinity was found to have increased over time, possibly due to the growing violence and geo-political conflicts in the region (consanguineous marriages are believed to strengthen pre-existing intra-familial relationships and thus be advantageous in the context of civil unrest) [21,22,23,24,25]. To our knowledge, no comprehensive study on the genetic spectrum of IRDs has ever been undertaken in North-Western Pakistan, possibly because of socio-economic and cultural limitations, a lack of infrastructure, difficult terrain, and escalating conflicts in the region.

## 2. Materials and Methods 

### 2.1. Enrollment of Families and Collection of Samples

Our study conforms to the standards of the Declaration of Helsinki and was approved by the Institutional Review Boards of the Hazara University, Mansehra, Pakistan (approval code: F.No:185/HU/Zool/2018/583) and of all our respective Institutions. Informed consents were provided in written form by all families prior to their participation and were signed by all members who were enrolled. Families with at least two or more affected persons, a history of consanguinity, and a clear autosomal recessive inheritance pattern of the disease were selected for molecular analysis. All families participating in our study were ethnically Pashtuns and were geographically located in the KP province in North-Western Pakistan. Clinical and demographic information was obtained via a pre-designed questionnaire, and pedigrees were drawn and cross-checked through face-to-face interviews with patients and/or elder members of the family, in their native language. Clinical data were obtained either directly from the patients’ medical reports (if available) or through consultation with the local clinicians/ophthalmologists who examined them on our request. Due to the limited medical infrastructure in the region, detailed clinical investigations, such as electroretinography (ERG) or fundus images, were not available. Furthermore, most of the clinical information obtained was derived from self-reported data, including, for example, difficulties in day/night vision, photophobia, disease onset and progression, response to medication, outcome of the Ishihara test, dark/light adaptation, ability to see/focus near and distant objects, loss of central or peripheral vision, and nystagmus. Patients were also investigated for other parameters, such as their ability to perform routine activities, e.g., reading, doing physical activities, and socializing with people. Electronic versions of pedigrees were created using the Pedigree Chart Designer (CeGaT, Tubingen, Germany). Saliva samples were collected by using the Oragene saliva kit (OG-500, DNA Genotek, Ottawa, ON, Canada) from patients as well as their clinically unaffected relatives, following the manufacturer’s guidelines. DNA was extracted from these samples following standard protocols, e.g., by following the prepIT-L2P manual (DNA Genotek, Canada). Quantitative and qualitative assessments of DNA were made using the NanoDrop™ 1000 Spectrophotometer (Thermo Fisher Scientific, Waltham, MA, USA) and electrophoresis on 1% agarose gels.

### 2.2. Genotyping and Homozygosity Mapping

Initially, nine families were subjected to genetic analysis using genome-wide high-density single-nucleotide polymorphism (SNP) arrays. For this purpose, genomic DNA of two or more individuals per family was genotyped by using the InfiniumCoreExome-24v1-1 array (Illumina, San Diego, CA, USA), which encompasses ~550,000 genome-wide SNP markers, at the iGE3 Genomics Platform of the University of Geneva, Switzerland. Arrays were processed using an iScan, according to the manufacturer’s protocol. Genotype calls were generated using the GenomeStudio software by Illumina. PLINK was used to analyze the genotype data [26]. Following the identification of shared autozygous intervals among two or more patients from the same family, all exons and exon–intron boundaries of candidate gene(s) inside these intervals were sequenced using the Sanger method. Additionally, families that belonged to the same geographic area and had clinically overlapping phenotypes were also investigated for the presence of shared autozygous intervals. Using this method, putative disease-causing variants were identified in four consanguineous pedigrees segregating autosomal recessive IRDs, while the remaining unsolved families were subsequently characterized by whole-exome sequencing (WES).

### 2.3. Whole-Exome Sequencing

Overall, WES was performed for 10 pedigrees. For WES analysis, 2.0 μg of genomic DNA from index patients was initially processed by Novogene Co. Ltd (Hong Kong, China). Sequencing libraries were generated using the Agilent SureSelect Human All ExonV6 kit (Agilent Technologies, Santa Clara, CA, USA), while fragmentation was carried out by hydrodynamic shearing (Covaris, Massachusetts, MA, USA). Following adapter ligation, DNA fragments were selectively enriched in a PCR reaction. Products were purified using the AMPure XP system (Beckman Coulter, Beverly, CA, USA) and quantified using an Agilent high-sensitivity DNA assay on the Agilent Bioanalyzer 2100 system. Captured DNA libraries underwent paired-end sequencing on an Illumina Novaseq 6000 S4 platform, resulting in sequences of 150 bases (PE150 sequencing strategy). WES data were analyzed using our in-house computational pipeline [27], and autozygosity mapping was done using AutoMap (unpublished). Finally, Sanger sequencing was performed to validate the potentially pathogenic variants detected and to confirm their causality, via genotype–phenotype cosegregation within the families.

## 3. Results

### 3.1. Clinical Synopsis

As mentioned earlier, detailed clinical investigation could not be achieved for all patients. However, a summary of the clinical information of one family is shown in Figure S1. On the basis of the few data available, mostly based on patients’ symptoms, we could identify five major clinical IRD classes. Briefly, patients with severe early-onset blindness were tentatively categorized as individuals with Leber congenital amaurosis (LCA, two families), while patients presenting with IRD and extra-ocular symptoms such obesity, hypogonadism, learning/developmental disabilities, post-axial polydactyly of hands and/or feet, and renal abnormalities were examined by a local clinician who classified them as suffering from Bardet–Biedl syndrome (BBS) (two families). Patients with progressive loss of central vision were categorized as having macular dystrophy (eleven families), while those presenting with initial night blindness and progressive loss of peripheral vision were classified as suffering from RP (four families). Patients with RP were clinically evaluated with the help of a local ophthalmologist who reported the presence of bilateral bone spicules and peripheral retinal vascular attenuation, through fundus examination. Lastly, patients with a complete inability to discriminate between colors were classified as having achromatopsia (one family).

### 3.2. Molecular Findings

**Figure 1:**
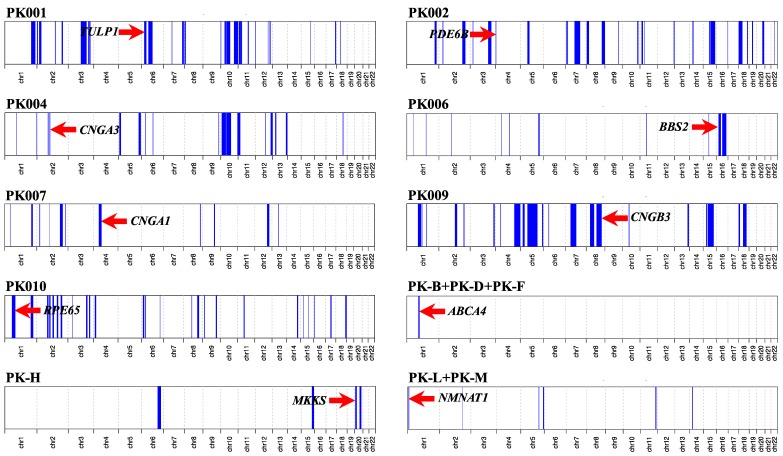
Homozygosity mapping showing genome-wide autozygous intervals as blue peaks. Red arrows indicate autozygous intervals harboring genes in which mutations were identified.

Collectively, we identified 11 disease-causing variants in 10 IRD-associated genes, in a total of 20 consanguineous IRDs pedigrees, all from North-Western Pakistan (Table 1). These variants were detected using a genome-wide SNP array followed by Sanger sequencing (four families), WES (ten families), and targeted Sanger sequencing alone (six families). Of these variants, seven were previously known IRD mutations, while four variants had never been identified before. Newly detected changes comprised three protein-truncating mutations and one nonsynonymous single-nucleotide variant (SNV). While the pathogenicity of protein-truncating variants can be easily postulated, the causality of the nonsynonymous SNV was inferred through in silico analysis and segregation studies. In spite of all families being consanguineous, compound heterozygosity was detected in one family (PK-E). All homozygous pathogenic variants were detected inside tractable autozygous intervals (≥2.0 Mb in size) and co-segregated with the disease in homozygosis in members from the remaining 19 families, including those who were not pre-ascertained by means of homozygosity mapping (Figure 1). Putative founder mutations were observed in the *ABCA4* (NM_000350.2:c.214G>A; p.Gly72Arg; 10 families) and in the *NMNAT1* genes (NM_022787.3:c.25G>A; p.Val9Met; 2 families), which together accounted for more than half of the IRD pedigrees analyzed in this study.

### 3.3. Macular Dystrophy (Possibly Including Stargardt Disease and Cone-Rod Degeneration)

**Figure 2:**
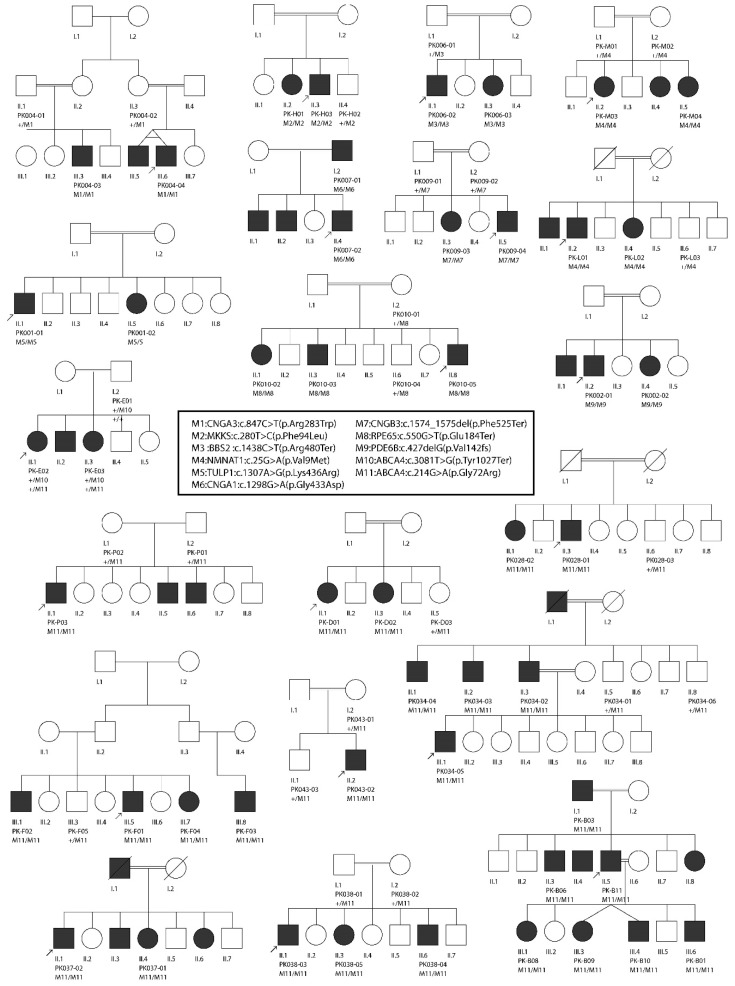
Segregation analysis of the mutations detected in a total of 20 consanguineous pedigrees from North-Western Pakistan. Probands in each pedigree are indicated by arrows. Due to space limitations, all pedigrees were trimmed.

Following SNP-based autozygosity mapping in three apparently unrelated families (PK-B, PK-D, and PK-F), we initially identified a ~2.0 Mb autozygous interval on chromosome 1, which was shared by three probands belonging to these families (Figure 1). Interestingly, the *ABCA4* gene was residing inside this interval, and an approximately 100 kb haplotype flanking this gene was identical in all three patients. WES analysis revealed a homozygous missense variant (NM_000350.2:c.214G>A:p.Gly72Arg) in *ABCA4*. The same variant (p.Gly72Arg) was also present in a compound heterozygous state with a nonsense mutation (NM_000350.2:c.3081T>G:p.Tyr1027Ter) in an additional family (PK-E) belonging to the same geographic location. Both of these variants have previously been identified to cause Stargardt disease [32,33]. Next, we performed targeted Sanger sequencing for p.Gly72Arg in a cohort of 18 previously uncharacterized consanguineous pedigrees from the region and identified the p.Gly72Arg mutation in six of them, in homozygosis. In total, p.Gly72Arg was found to cause disease in at least 10 independent pedigrees from a small town in North-Western Pakistan, collectively accounting for 37 patients (Figure 2). Geographically, these families belonged to Darra Adam Khel in North-Western Pakistan, an area which is mainly inhabited by the Afridi clan of Pashtuns ethnicity. Since these families were from the same geographic region and had a common ethnic affiliation, and an identical haplotype around *ABCA4* was detected in the three patients who were investigated for it, we believe that p.Gly72Arg constitutes a founder mutation.

Furthermore, WES analysis in family PK009 revealed a nonsense variant (NM_019098.4:c.1574_1575del:p.Phe525Ter) in the *CNGB3* gene, which co-segregated with the disease in homozygosis (Figure 2). This variant has never been reported in any public databases and constitutes a loss-of-function allele, therefore likely representing the molecular cause of disease in this family.

### 3.4. Retinitis Pigmentosa

Through whole-exome sequencing in four consanguineous families with autosomal recessive RP, we identified disease-causing variants in four IRD-associated genes. These included: *TULP1* (NM_001289395.1:c.1307A>G:p.Lys436Arg; family PK001), *PDE6B* (NM_001145291.1:c.427del:p.Ala143LeufsTer7; family PK002), *CNGA1* (NM_001142564.1: c.1298G>A:p.Gly433Asp; family PK007), and *RPE65* (NM_000329.2:c.550G>T:p.Glu184Ter; family PK010). All genes harboring these variants were located inside autozygous intervals with a genomic size of ≥2 Mb (Figure 1). All variants were validated by Sanger sequencing and confirmed to co-segregate with the disease in all affected family members (Figure 2). While variants identified in the *TULP1* (NM_001289395.1:c.1307A>G:p.Lys436Arg) and in the *CNGA1* (NM_001142564.1: c.1298G>A:p.Gly433Asp) genes were formerly known to cause RP [18,31], DNA changes in *PDE6B* and in *RPE65* have never been reported in any public databases. Since they are both loss-of-function variants (nonsense and frame-shift variants), they can be assumed to represent bona fide mutations.

### 3.5. Leber Congenital Amaurosis (Early-Onset Retinal Blindness)

Using SNP-based autozygosity mapping in two families with early-onset visual problems (PK-L, PK-M), we identified an autozygous interval on chromosome 1 that was shared by both probands from these families (Figure 1). Since *NMNAT1*, residing in this region, was a suitable candidate gene for LCA, we screened all exons and exon–intron boundaries of this gene, using Sanger sequencing. We found a missense variant (NM_022787.3:c.25G>A:p.Val9Met) in exon 2 that co-segregated with the disease in both families (Figure 2). The same variant (p.Val9Met) was previously reported to cause LCA in a pedigree of Pakistani descent [30]. However, we could not establish whether this previously identified family had any relationship with the pedigrees analyzed in our study. Considering the geographic proximity of these families, we suggest that p.Val9Met in *NMNAT1* constitutes another example of a founder mutation in North-Western Pakistan.

### 3.6. Bardet–Biedl Syndrome

Again, autozygosity mapping identified common intervals on chromosomes 16 and 20 in two families (PK006 and PK-H) with BBS (Figure 1); these intervals included the *BBS2* and the *MKKS* genes, respectively. In affected members of family PK006, the previously reported homozygous nonsense mutation *BBS2*:NM_031885.3:c.1438C>T:p.Arg480Ter was found [29], whereas family PK-H segregated a novel missense mutation (*MKKS*:NM_170784.2:c.280T>C:p.Phe94Leu). Both *BBS2* and *MKKS* variants co-segregated with the disease in a homozygous state and were present heterozygously in healthy individuals from both families (Figure 2). The *MKKS* variant was found to result in the disruption of a highly conserved amino acid (Phe94) and was predicted to be highly deleterious by numerous online prediction tools such as PolyPhen-2, PROVEAN, Mutation Taster, SIFT, MutationTaster2, and LRT [34,35,36,37,38,39]. Additionally, the variant was never identified in any public databases, including The Genome Aggregation Database (gnomAD), The Exome Aggregation Consortium (ExAC), and The Human Gene Mutation Database (HGMD) [40,41,42].

### 3.7. Achromatopsia

Through exome sequencing in a consanguineous pedigree (PK004) with three affected children suffering from putative complete achromatopsia, we identified a homozygous nonsynonymous single-nucleotide variant (NM_001298.2:c.847C>T:p.Arg283Trp) in the *CNGA3* gene. Autozygosity mapping revealed the *CNGA3* gene to lie within a 5 Mb autozygous interval on chromosome 2 (Figure 1). The mutation is a known cause of achromatopsia [28].

## 4. Discussion

Pakistan has one of the highest prevalence of inherited genetic diseases in the world [13], likely due to the high consanguinity rate of its population, generally exceeding 50% [11,12,14]. In this country, marriages of first cousins are highly favored, and families from Pakistan are considered a valuable resource for medical genetics research, which has led to significant scientific findings in the recent past [13,43]. Several studies on IRDs have been conducted in Pakistan during the last few years, but the majority of them were based on pedigrees from the Punjab and Sindh provinces [17,18,19,20]. Khan et al. [20] showed that 90% of the mutations for non-syndromic IRDs and 100% of the mutations for syndromic IRDs were specific to families of Pakistani origin and that mutations in 35 different genes were found to cause non-syndromic IRDs specifically in families of Pakistani descent. In our study, we observed a different trend: out of the 11 mutations identified, only 4 were novel, whereas the remainder had previously been reported in patients from China, the UK, and Germany, and only two of them were found in Pakistani residents [28,29,31,32,33]. This probably reflects the fact that we focused our analysis on pedigrees from the North-Western part of the country, which remains largely isolated from populations inhabiting central Pakistan for cultural, linguistic, and geographic reasons. Further, North-Western Pakistan is predominantly a Pashtuns territory and, in contrast to central Pakistan, these populations trace their lineage to Pashtuns living in Western Afghanistan, with whom they share, even today, linguistic and sociocultural affiliations. 

Our data therefore provide a first insight into the genetic landscape of IRDs in this peculiar part of the world, slightly extending the known mutational spectrum of IRDs. In particular, we have reported two founder mutations (*ABCA4*:c.214G>A:p.Gly72Arg and *NMNAT1*:c.25G>A:p.Val9Met) that, together, were responsible for disease in more than half of the patients in our study. At least 37 patients from 10 different families were found to be affected by the mutation in *ABCA4*, while the *NMNAT1* mutation touched at least 6 people in two unrelated families. Owing to the high rates of traditional intra-familial marriages, strong socio-cultural and ethnic divides, as well as geographic barriers, we predict that the number of patients/families affected by these two founder mutations may be even higher. Furthermore, a considerable number of variants identified in our study were detected inside large autozygous intervals (≥10 Mb), thus reflecting recent endogamy in the population. Similarly, our data support the previous notion that the likelihood for a homozygous pathogenic variant to be found within one autozygous interval is much higher in consanguineous pedigrees [10,44,45,46].

In summary, our study explored the genetic landscape of inherited retinal diseases in North-Western Pakistan, a large but relatively ignored part of the country. In addition to detecting a high degree of autozygosity and relevant founder mutations in the region, our data further expand the mutational spectrum of IRD-associated genes by adding four new variants to it. These findings will help future researchers/clinicians in their rapid screenings of patients from this region and will assist families seeking genetic counselling. We also predict that these insights on eye disorders might apply to other rare conditions that affect individuals from this region, such as deafness, intellectual disabilities, and developmental defects.

## Figures and Tables

**Table 1:** Molecular findings in 20 consanguineous pedigrees from North-Western Pakistan segregating autosomal recessive inherited retinal dystrophies (IRDs).

Family ID	Tentative Diagnosis	Gene Name	Transcript ID	cDNA Change	Protein Change	GnomAD MAF	Mutation Number	Zyg-Osity	ROH Size	Method	References
PK004	ACHM	*CNGA3*	NM_001298.2	c.847C>T	p.Arg283Trp	0.0001402	M1	Hom	5-Mb	WES	[28]
PK-H	BBS	*MKKS*	NM_170784.2	c.280T>C	p.Phe94Leu	NA	M2	Hom	10-Mb	SNP array	This study
PK006	BBS	*BBS2*	NM_031885.3	c.1438C>T	p.Arg480Ter	0.00001647	M3	Hom	31-Mb	SNP array	[29]
PK-L	LCA	*NMNAT1*	NM_022787.3	c.25G>A	p.Val9Met	NA	M4	Hom	2-Mb	SNP array	[30]
PK-M	LCA	*NMNAT1*	NM_022787.3	c.25G>A	p.Val9Met	NA	M4	Hom	2-Mb	SNP array	[30]
PK001	RP	*TULP1*	NM_001289395.1	c.1307A>G	p.Lys436Arg	0.00002472	M5	Hom	17-Mb	WES	[31]
PK007	RP	*CNGA1*	NM_001142564.1	c.1298G>A	p.Gly433Asp	NA	M6	Hom	21-Mb	WES	[18]
PK009	MD	*CNGB3*	NM_019098.4	c.1574_1575del	p.Phe525Ter	NA	M7	Hom	39-Mb	WES	This study
PK010	RP	*RPE65*	NM_000329.2	c.550G>T	p.Glu184Ter	NA	M8	Hom	21-Mb	WES	This study
PK002	RP	*PDE6B*	NM_001145291.1	c.427del	p.Ala143LeufsTer7	NA	M9	Hom	4-Mb	WES	This study
PK-E	MD	*ABCA4*	NM_000350.2	c.3081T>G	p.Tyr1027Ter	NA	M10	Het	NA	SNP-WES	[32]
PK-E	MD	*ABCA4*	NM_000350.2	c.214G>A	p.Gly72Arg	0.00002784	M11	Het	NA	SNP-WES	[33]
PK-B	MD	*ABCA4*	NM_000350.2	c.214G>A	p.Gly72Arg	0.00002784	M11	Hom	7-Mb	SNP-WES	[33]
PK-P	MD	*ABCA4*	NM_000350.2	c.214G>A	p.Gly72Arg	0.00002784	M11	Hom	NA	TSS	[33]
PK-F	MD	*ABCA4*	NM_000350.2	c.214G>A	p.Gly72Arg	0.00002784	M11	Hom	24-Mb	SNP-WES	[33]
PK-D	MD	*ABCA4*	NM_000350.2	c.214G>A	p.Gly72Arg	0.00002784	M11	Hom	24-Mb	SNP-WES	[33]
PK028	MD	*ABCA4*	NM_000350.2	c.214G>A	p.Gly72Arg	0.00002784	M11	Hom	NA	TSS	[33]
PK034	MD	*ABCA4*	NM_000350.2	c.214G>A	p.Gly72Arg	0.00002784	M11	Hom	NA	TSS	[33]
PK037	MD	*ABCA4*	NM_000350.2	c.214G>A	p.Gly72Arg	0.00002784	M11	Hom	NA	TSS	[33]
PK038	MD	*ABCA4*	NM_000350.2	c.214G>A	p.Gly72Arg	0.00002784	M11	Hom	NA	TSS	[33]
PK043	MD	*ABCA4*	NM_000350.2	c.214G>A	p.Gly72Arg	0.00002784	M11	Hom	NA	TSS	[33]

MAF: Minor allele frequency; NA: Not available; Hom: Homozygous; Het: Heterozygous; ROH: Runs of homozygosity; WES: Whole-exome sequencing; TSS: Targeted Sanger sequencing. ACHM: Achromatopsia; BBS: Bardet–Biedl syndrome; LCA: Leber congenital amaurosis; RP: Retinitis pigmentosa; MD: Macular dystrophy.

## Supplementary Materials

The following are available online at https://www.mdpi.com/2073-4425/11/1/12/s1, Figure S1: Clinical features from the proband of family PK-H (*MKKS*:NM_170784.2:c.280T>C:p.Phe94Leu). Fundus images of the patient’s right (A) and left (B) eye. Post-axial polydactyly of the right (C) and left (D) foot, indicated by red arrows.

## References

[1] Hamel C.P. (2014). Gene discovery and prevalence in inherited retinal dystrophies. C. R. Biol..

[2] Berger W., Kloeckener-Gruissem B., Neidhardt J. (2010). The molecular basis of human retinal and vitreoretinal diseases. Prog. Retin. Res.

[3] Duncan J.L., Pierce E.A., Laster A.M., Daiger S.P., Birch D.G., Ash J.D., Iannaccone A., Flannery J.G., Sahel J.A., Zack D.J. (2018). Inherited Retinal degenerations: Current Landscape and knowledge gaps. Transl. Vis. Sci. Technol..

[4] Brown M.D., Voljavec A.S., Lott M.T., MacDonald I., Wallace D.C. (1992). Leber’s hereditary optic neuropathy: A model for mitochondrial neurodegenerative diseases. Faseb. J..

[5] Traboulsi E.I. (2009). Hope and major strides for genetic diseases of the eye. J. Genet..

[6] Stone E.M., Andorf J.L., Whitmore S.S., DeLuca A.P., Giacalone J.C., Streb L.M., Braun T.A., Mullins R.F., Scheetz T.E., Sheffield V.C. (2017). Clinically focused molecular investigation of 1000 consecutive families with inherited retinal disease. Ophthalmology.

[7] Abu-Safieh L., Alrashed M., Anazi S., Alkuraya H., Khan A.O., Al-Owain M., Al-Zahrani J., Al-Abdi L., Hashem M., Al-Tarimi S. (2013). Autozygome-guided exome sequencing in retinal dystrophy patients reveals pathogenetic mutations and novel candidate disease genes. Genome Res..

[8] Khalak H.G., Wakil S.M., Imtiaz F., Ramzan K., Baz B., Almostafa A., Hagos S., Alzahrani F., Abu-Dhaim N., Abu Safieh L. (2012). Autozygome maps dispensable DNA and reveals potential selective bias against nullizygosity. Genet. Med..

[9] Alkuraya F.S. (2010). Autozygome decoded. Genet. Med..

[10] Alkuraya F.S. (2013). The application of next-generation sequencing in the autozygosity mapping of human recessive diseases. Hum. Genet..

[11] Chinthapalli K. (2013). First cousin marriage can double risk of birth defects, finds study. Br. Med. J..

[12] Hamamy H., Antonarakis S.E., Cavalli-Sforza L.L., Temtamy S., Romeo G., Kate L.P., Bennett R.L., Shaw A., Megarbane A., van Duijn C. (2011). Consanguineous marriages, pearls and perils: Geneva international consanguinity workshop report. Genet. Med..

[13] Riaz M., Tiller J., Ajmal M., Azam M., Qamar R., Lacaze P. (2019). Implementation of public health genomics in Pakistan. Eur. J. Hum. Genet..

[14] Pakistan I., National Institute of Population Studies (2013). Pakistan Demographic and Health Survey.

[15] Hassan B., Ahmed R., Li B., Noor A., Hassan Z.U. (2019). A comprehensive study capturing vision loss burden in Pakistan (1990–2025): Findings from the global burden of disease (GBD) 2017 study. PLoS ONE.

[16] Adhi M.I., Ahmed J. (2002). Frequency and clinical presentation of retinaly dystrophies—A Hospital based study. Pak. J. Ophthalmol..

[17] Li L., Chen Y., Jiao X., Jin C., Jiang D., Tanwar M., Ma Z., Huang L., Ma X., Sun W. (2017). Homozygosity mapping and genetic analysis of autosomal recessive retinal dystrophies in 144 consanguineous pakistani families. Invest. Ophthalmol. Vis. Sci..

[18] Maria M., Ajmal M., Azam M., Waheed N.K., Siddiqui S.N., Mustafa B., Ayub H., Ali L., Ahmad S., Micheal S. (2015). Homozygosity mapping and targeted sanger sequencing reveal genetic defects underlying inherited retinal disease in families from pakistan. PLoS ONE.

[19] Maranhao B., Biswas P., Gottsch A.D., Navani M., Naeem M.A., Suk J., Chu J., Khan S.N., Poleman R., Akram J. (2015). Investigating the molecular basis of retinal degeneration in a familial cohort of pakistani decent by exome sequencing. PLoS ONE.

[20] Khan M.I., Azam M., Ajmal M., Collin R.W., den Hollander A.I., Cremers F.P., Qamar R. (2014). The molecular basis of retinal dystrophies in pakistan. Genes.

[21] Pervaiz R., Faisal F., Serakinci N. (2018). Practice of consanguinity and attitudes towards risk in the pashtun population of khyber pakhtunkhwa, pakistan. J. Biosoc. Sci..

[22] Ahmad B., Rehman A.U., Malik S. (2016). Consanguinity and inbreeding coefficient in tribal pashtuns inhabiting the turbulent and war-affected territory of bajaur agency, north-west pakistan. J. Biosoc. Sci..

[23] Rehman A.U., Ahmad I., Zaman M., Malik S. (2016). Transition in consanguinity in dir lower district, a victim of war, natural disaster and population displacement, in north-west pakistan—A response to sthanadar et al. (2015). J. Biosoc. Sci..

[24] Sthanadar A.A., Bittles A.H., Zahid M. (2014). Civil unrest and the current profile of consanguineous marriage in Khyber Pakhtunkhwa province, Pakistan. J. Biosoc. Sci..

[25] Sthanadar A.A., Bittles A.H., Zahid M. (2016). Increasing prevalence of consanguineous marriage confirmed in Khyber Pakhtunkhwa province, Pakistan. J. Biosoc. Sci..

[26] Purcell S., Neale B., Todd-Brown K., Thomas L., Ferreira M.A., Bender D., Maller J., Sklar P., de Bakker P.I., Daly M.J. (2007). PLINK: A tool set for whole-genome association and population-based linkage analyses. Am. J. Hum. Genet..

[27] Royer-Bertrand B., Castillo-Taucher S., Moreno-Salinas R., Cho T.J., Chae J.H., Choi M., Kim O.H., Dikoglu E., Campos-Xavier B., Girardi E. (2015). Mutations in the heat-shock protein A9 (HSPA9) gene cause the EVEN-PLUS syndrome of congenital malformations and skeletal dysplasia. Sci. Rep..

[28] Kohl S., Marx T., Giddings I., Jagle H., Jacobson S.G., Apfelstedt-Sylla E., Zrenner E., Sharpe L.T., Wissinger B. (1998). Total colourblindness is caused by mutations in the gene encoding the alpha-subunit of the cone photoreceptor cGMP-gated cation channel. Nat. Genet..

[29] Xing D.J., Zhang H.X., Huang N., Wu K.C., Huang X.F., Huang F., Tong Y., Pang C.P., Qu J., Jin Z.B. (2014). Comprehensive molecular diagnosis of Bardet-Biedl syndrome by high-throughput targeted exome sequencing. PLoS ONE.

[30] Falk M.J., Zhang Q., Nakamaru-Ogiso E., Kannabiran C., Fonseca-Kelly Z., Chakarova C., Audo I., Mackay D.S., Zeitz C., Borman A.D. (2012). NMNAT1 mutations cause Leber congenital amaurosis. Nat. Genet..

[31] Gu S., Lennon A., Li Y., Lorenz B., Fossarello M., North M., Gal A., Wright A. (1998). Tubby-like protein-1 mutations in autosomal recessive retinitis pigmentosa. Lancet.

[32] Fujinami K., Zernant J., Chana R.K., Wright G.A., Tsunoda K., Ozawa Y., Tsubota K., Robson A.G., Holder G.E., Allikmets R. (2015). Clinical and molecular characteristics of childhood-onset Stargardt disease. Ophthalmology.

[33] Rivera A., White K., Stohr H., Steiner K., Hemmrich N., Grimm T., Jurklies B., Lorenz B., Scholl H.P., Apfelstedt-Sylla E. (2000). A comprehensive survey of sequence variation in the ABCA4 (ABCR) gene in Stargardt disease and age-related macular degeneration. Am. J. Hum. Genet..

[34] Adzhubei I., Jordan D.M., Sunyaev S.R. (2013). Predicting functional effect of human missense mutations using PolyPhen-2. Curr. Protoc. Hum. Genet..

[35] Choi Y., Chan A.P. (2015). PROVEAN web server: A tool to predict the functional effect of amino acid substitutions and indels. Bioinformatics.

[36] Schwarz J.M., Rodelsperger C., Schuelke M., Seelow D. (2010). MutationTaster evaluates disease-causing potential of sequence alterations. Nat. Methods.

[37] Sim N.L., Kumar P., Hu J., Henikoff S., Schneider G., Ng P.C. (2012). SIFT web server: Predicting effects of amino acid substitutions on proteins. Nucleic Acids Res..

[38] Schwarz J.M., Cooper D.N., Schuelke M., Seelow D. (2014). MutationTaster2: Mutation prediction for the deep-sequencing age. Nat. Methods.

[39] Chun S., Fay J.C. (2009). Identification of deleterious mutations within three human genomes. Genome Res..

[40] Stenson P.D., Ball E.V., Mort M., Phillips A.D., Shiel J.A., Thomas N.S., Abeysinghe S., Krawczak M., Cooper D.N. (2003). Human gene mutation database (HGMD): 2003 update. Hum. Mutat..

[41] Lek M., Karczewski K.J., Minikel E.V., Samocha K.E., Banks E., Fennell T., O’Donnell-Luria A.H., Ware J.S., Hill A.J., Cummings B.B. (2016). Analysis of protein-coding genetic variation in 60,706 humans. Nature.

[42] Karczewski K.J., Francioli L.C., Tiao G., Cummings B.B., Alföldi J., Wang Q., Collins R.L., Laricchia K.M., Ganna A., Birnbaum D.P. (2019). Variation across 141, 456 human exomes and genomes reveals the spectrum of loss-of-function intolerance across human protein-coding genes. BioRxiv.

[43] Saleheen D., Natarajan P., Armean I.M., Zhao W., Rasheed A., Khetarpal S.A., Won H.H., Karczewski K.J., O’Donnell-Luria A.H., Samocha K.E. (2017). Human knockouts and phenotypic analysis in a cohort with a high rate of consanguinity. Nature.

[44] Wakeling M.N., Laver T.W., Wright C.F., De Franco E., Stals K.L., Patch A.M., Hattersley A.T., Flanagan S.E., Ellard S., Study D.D.D. (2019). Homozygosity mapping provides supporting evidence of pathogenicity in recessive Mendelian disease. Genet. Med..

[45] Szpiech Z.A., Xu J., Pemberton T.J., Peng W., Zollner S., Rosenberg N.A., Li J.Z. (2013). Long runs of homozygosity are enriched for deleterious variation. Am. J. Hum. Genet..

[46] Alkuraya F.S. (2012). Discovery of rare homozygous mutations from studies of consanguineous pedigrees. Curr. Protoc. Hum. Genet..

