# Supplementary figures and images for: Exploring the Genetic Landscape of Retinal Diseases in North-Western Pakistan Reveals a High Degree of Autozygosity and a Prevalent Founder Mutation in ABCA4
Source: Genes (Basel). 2019 Dec 21;11(1):12. doi: 10.3390/genes11010012 (PMC7017091; doi:10.3390/genes11010012)

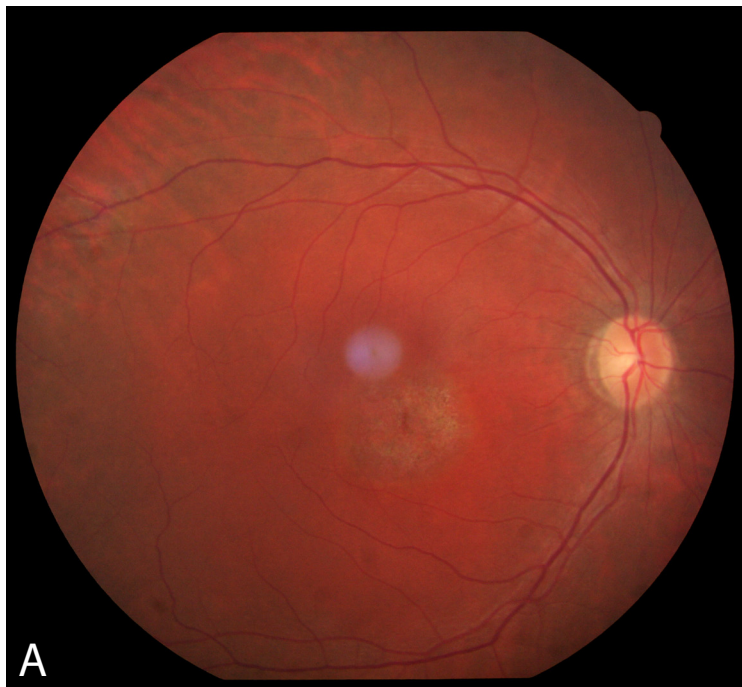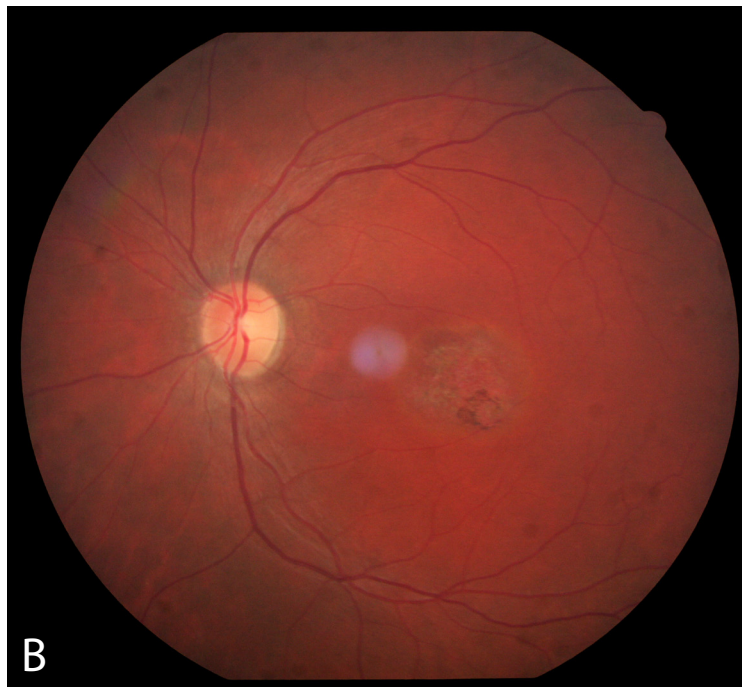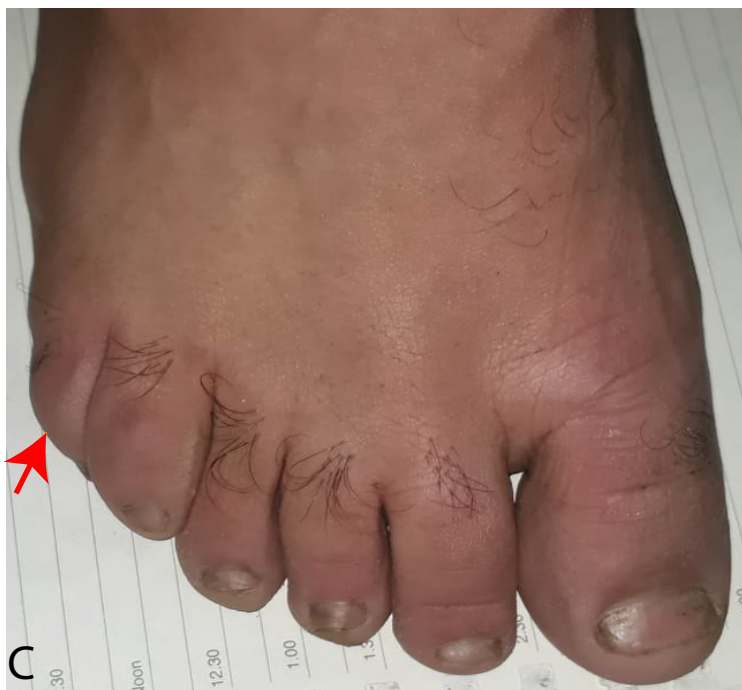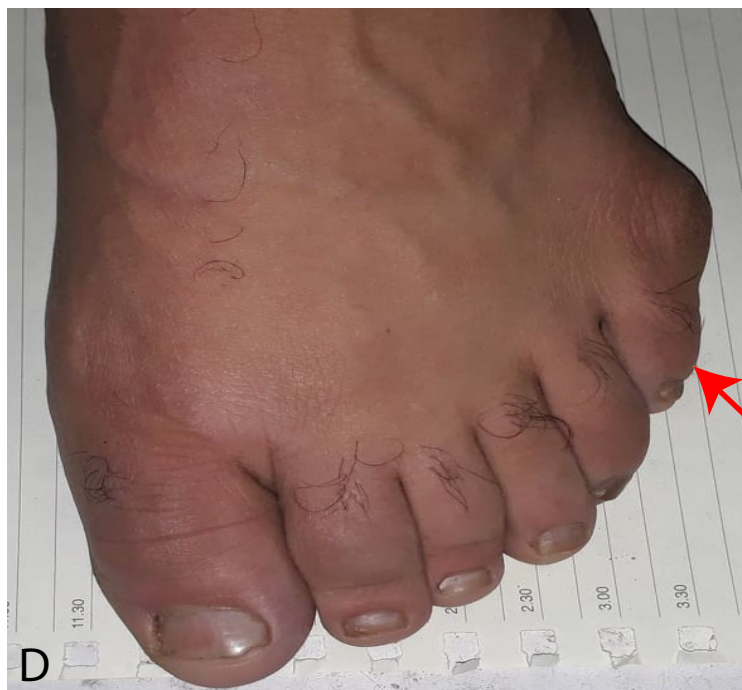

Supplement: Supplementary file 1 [file genes-11-00012-s001.pdf]
